# Global changes in mineral transporters in tetraploid switchgrasses (*Panicum virgatum* L.)

**DOI:** 10.3389/fpls.2013.00549

**Published:** 2014-01-02

**Authors:** Nathan A. Palmer, Aaron J. Saathoff, Brian M. Waters, Teresa Donze, Tiffany M. Heng-Moss, Paul Twigg, Christian M. Tobias, Gautam Sarath

**Affiliations:** ^1^USDA-Agricultural Research Service, Grain, Forage, and Bioenergy Research UnitLincoln, NE, USA; ^2^Department of Agronomy and Horticulture, University of NebraskaLincoln, NE, USA; ^3^LI-COR BiosciencesLincoln, NE, USA; ^4^Department of Entomology, University of NebraskaLincoln, NE, USA; ^5^Biology Department, University of NebraskaKearney, NE, USA; ^6^USDA-Agricultural Research Service, Genomics and Gene Discovery Research Unit, Western Regional Research CenterAlbany, CA, USA

**Keywords:** bioenergy, crowns and rhizomes, growing-season, mineral transporters, nutrients, *Panicum virgatum*, switchgrass, qPCR

## Abstract

Switchgrass (*Panicum virgatum L*) is perennial, C_4_ grass with great potential as a biofuel crop. An in-depth understanding of the mechanisms that control mineral uptake, distribution and remobilization will benefit sustainable production. Nutrients are mobilized from aerial portions to below-ground crowns and rhizomes as a natural accompaniment to above-ground senescence post seed-set. Mineral uptake and remobilization is dependent on transporters, however, little if any information is available about the specific transporters that are needed and how their relative expression changes over a growing season. Using well-defined classes of mineral transporters, we identified 520 genes belonging to 40 different transporter classes in the tetraploid switchgrass genome. Expression patterns were determined for many of these genes using publically available transcriptomic datasets obtained from both greenhouse and field grown plants. Certain transporters showed strong temporal patterns of expression in distinct developmental stages of the plant. Gene-expression was verified for selected transporters using qRT-PCR. By and large these analyses confirmed the developmental stage-specific expression of these genes. Mineral analyses indicated that K, Fe, Mg, Co, and As had a similar pattern of accumulation with apparent limited remobilization at the end of the growing season. These initial analyses will serve as a foundation for more detailed examination of the nutrient biology of switchgrass.

## Introduction

Plant mineral composition depends on uptake and translocation of minerals from the rhizosphere, through the root-shoot junction (crown), and into the aboveground tissues. These processes are influenced by both environmental and genotypic factors. In the perennial plant growth cycle, certain minerals can be recycled or remobilized from senescing tissues in the autumn, stored in perennial tissues during winter dormancy, and then remobilized and translocated to growing tissues in the spring. In switchgrass (*Panicum virgatum* L.), the stems and leaves survive for 1 year, while perennial tissues including the crowns, rhizomes, and older roots survive for much longer periods of time. The crown and rhizome tissues connect the root system to the shoot system, thus, minerals that are remobilized from annual or perennial tissues must pass through the crown tissue. These tissues can also serve as repository for remobilized nutrients at the end of the growing season. Thus, mineral uptake and recycling are cornerstones for the sustainable production of biomass from switchgrass and other perennial herbaceous bioenergy crops.

In *Arabidopsis thaliana*, concentrations of several minerals were highest in young tissues (Waters and Grusak, 2008), suggesting that stage of harvest may be important for mineral concentration in plant tissues. In switchgrass, harvests during late vegetative stage or during summer had substantially higher P, Cl, K, and S than at post-senescence stage (Reynolds et al., 2000; Vogel et al., 2002; Dien et al., 2006; Lemus et al., 2009; Yang et al., 2009), demonstrating that these compounds are remobilized from leaf and stem tissue. Genotypic differences in remobilization were shown to be present (Reynolds et al., 2000; El-Nashaar et al., 2009; Yang et al., 2009).

The most abundant minerals in above ground switchgrass tissues are Si, K, Cl, Ca, and P (Monti et al., 2008; El-Nashaar et al., 2009). While several minerals are remobilized from aboveground biomass during senescence in switchgrass, some abundant minerals are not, such as Si, Ca, and Mg (Dien et al., 2006; Lemus et al., 2009; Yang et al., 2009). Feedstock quality requirements depend on the conversion platform (Sarath et al., 2008; Vogel et al., 2011) and pyrolysis and other thermochemical platforms will benefit from feedstocks that contain high lignin and low levels of N and alkali metals (Patwardhan et al., 2010). Reducing minerals such as Si will lower ash content as well. A number of genes that contribute to root uptake of Ca and Mg (Karley and White, 2009; Waters, 2011), and transporters for Si uptake and distribution (Ma et al., 2006; Yamaji et al., 2008; Yamaji and Ma, 2009) have been identified in grasses. Currently, no whole genome-scale annotation and transcriptomic information for mineral and related transporter genes are available in switchgrass, however, this data would be useful to correlate specific genes with mineral accumulation.

Increased understanding of genes that impact mineral acquisition, transport and recycling in switchgrass can be used to improve both the genetics and management of switchgrass as a high-yielding biomass crop. Mineral transporters will be key players in these processes, as transport into and out of cells and organelles are the molecular events that underlie cellular storage and whole-plant translocation or recycling of minerals. Mineral transporter families have been studied extensively in species such as Arabidopsis and rice, providing gene sequence data to predict identity and function of unknown transcripts from other species. In switchgrass, no molecular studies of transporter genes have been conducted. Understanding of the interactions in uptake and remobilization between different minerals over the course of a growing season is limited for switchgrass. The release of the switchgrass genome (PviDraft0.0) by the Joint Genomes Institute (www.phytozome.org) (Goodstein et al., 2012) has greatly facilitated the discovery and annotation of genes and gene families in switchgrass (Saathoff et al., 2013).

The long-term goal of our research is to develop and utilize genotyping and phenotyping tools that can significantly enhance the breeding of switchgrass (*Panicum virgatum* L.) as a sustainable bioenergy crop for marginal crop lands (Vogel et al., 2011). Our objectives in this study were to use next-generation sequencing data to discover, annotate and quantify expression of switchgrass genes that are potentially involved with mineral transport in switchgrass. Here, we have combined bioinformatics and real-time qRT-PCR to classify transporter gene families in switchgrass and to identify specific genes that show altered expression over the growing season. We also used mineral analysis to quantify seasonal concentration changes in crown tissues.

## Materials and methods

### Gene discovery

Known mineral and nutrient transporters in *Arabidopsis thaliana* were used to identify putative homologs in *Panicum virgatum, Sorghum bicolor*, and *Setaria italica* based on protein similarity using BLASTp (Altschul et al., 1990, 1997) and the respective reference genomes for each plant (www.phytozome.org) (Paterson et al., 2009; Bennetzen et al., 2012; Goodstein et al., 2012). A maximum *e*-value of 1 × 10^−25^ and minimum alignment of 50% were used as thresholds in filtering the BLASTp results for putative homolog identification.

### Phylogenetic analyses

Cladograms were generated for genes of selected Arabidopsis and putative switchgrass transporter families. Sequences were analyzed for phylogenetic relationships using Phylogeny.fr (Dereeper et al., 2008).

Publically available 454 transcriptome sequencing datasets were used to generate expression profiles for identified putative mineral and nutrient transporters in switchgrass as described previously (Saathoff et al., 2013). 454 sequence reads were aligned to the draft switchgrass transcriptome using Bowtie2 (Langmead and Salzberg, 2012) and gene counts calculated using HTSeq-Count version 0.5.1p2 (https://pypi.python.org/pypi/HTSeq). Expression counts were normalized through conversion from raw counts to RPKM (reads per kilo base exon per million mapped reads).

### Heat maps and clustering

Heat maps were generated using estimated 454 expression data and two-way hierarchical clustering with JMP 9.0 (SAS Institute Inc., Cary, NC). RPKM expression values were converted to standardized values (z-scores) for each gene, and hierarchical clustering using Ward's method was performed to yield heat maps and clusters of coexpressing transporters.

### Plant material

Crowns and rhizomes were collected, cleaned and flash-frozen from field-established plants of cv Summer, as described earlier (Palmer et al., 2012). At each harvest date tissues were obtained from three individual plants. Flash-frozen tissues were stored at −80°C until needed. Tissues were ground in a cryogenic grinder (Palmer et al., 2012). Aliquots (0.1 g) of ground materials were used for isolating RNA as previously described (Chomczynski and Sacchi, 1987). RNA samples were subsequently purified using RNeasy columns (Qiagen; Valencia, CA, USA) according to manufacturer's instructions.

### qRT-PCR and primers

DNase treated RNA samples were used to synthesize first strand cDNA by using SuperScript III reverse transcriptase (Invitrogen; Carlsbad, CA, USA) and random primers according to the manufacturer's protocol. qRT-PCR reactions were set up in a total volume of 15 uL using 7.5 uL master mix (Bio-Rad), 0.2 uL cDNA template, 0.75 uL primers, and 6.55 uL 18 MΩ water and conducted on a BioRAD CFX Connect Real Time PCR instrument. Each reaction was performed in quadruplicate and the experimental design blocked plate with amplicon (a single amplicon per plate). Primers were designed using Primer3Plus (Untergasser et al., 2012). Data was efficiency corrected using LinRegPCR (Ramakers et al., 2003; Ruijter et al., 2009), and geNORM was used to screen for effective normalization genes and calculate relative quantities for each gene of interest (Hellemans et al., 2007). Primers used are shown in Table A1.

### Mineral analyses

Tissues were dried at 60°C for at least 72 h and weighed. Samples (typically 25–50 mg) were digested as described previously (Waters et al., 2012). In brief, samples were digested with 3 ml of concentrated HNO_3_ (VWR, West Chester, PA, USA, Trace metal grade) at room temperature overnight, then at 100°C for 1.5 h, followed by addition of 2 ml of 30% H_2_O_2_ (Fisher Scientific, Fair Lawn, NJ, USA) and digestion for 1 h each at steps of 125°C, 150°C, 165°C, and finally were heated to dryness at 180°C. Dried samples were then resuspended in 5 ml of 1% HNO3, and minerals were quantified by inductively coupled plasma mass spectrometry (ICP-MS) at the University of Nebraska Redox Biology Center Spectroscopy and Biophysics Core Facility.

### Statistical analyses

Transcript levels were investigated by utilizing cDNA that originated from three individual genotypes (biological replicates) at each time point with four technical replicates per genotype. The cDNA was not pooled prior to qRT-PCR analysis. Thus, for any given harvest date, there were 12 total reactions that were conducted which included both true biological as well as technical replicates. The GeNORM program (Hellemans et al., 2007) within the qbase+ software package was used to analyze reference genes in order to find suitably stable ones with a *M*-value below 1.5. In this way, Pavirv00026367m (a ubiquitin protein ligase) was selected as the stable reference gene for generation of relative quantities. The relative quantities were then statistically analyzed using PROC GLM in SAS (SAS Institute, Cary, NC) and Tukey's multiple comparison procedure was utilized to conduct pairwise comparisons of different harvest dates. Familywise error rate was controlled at α = 0.05.

Data for the mineral analyses were subjected to single-factor ANOVA analysis of each mineral, error bars are standard deviations are from 3 biological replicates, with 2 technical replicates each. Of the 16 minerals analyzed, the 10 minerals showed statistically significant variation for at least two time points. *P*-values for the mineral analysis were calculated by Single Factor ANOVAs (in Excel).

## Results and discussion

Little is known about the identities of specific genes that contribute to remobilization of minerals from senescing tissues. Some genes are known to be important for remobilization and/or translocation of minerals from source to sink tissues in Arabidopsis, for example *YSL1, YSL3*, and *OPT3* for iron, zinc, and copper (Waters et al., 2006; Stacey et al., 2008; Waters and Grusak, 2008), *NRT1.7* for nitrate (Fan et al., 2009), *Sultr1;3* for sulfate (Yoshimoto et al., 2003), and *Pht1;5* for phosphate (Nagarajan et al., 2011). These genes have usually been discovered by analysis of mutants. A transcriptomic approach can reveal new insights to help understand nutrient deficiency signaling pathways (Maruyama-Nakashita et al., 2003; Hermans et al., 2010; Waters et al., 2012). Likewise, transcriptomic studies in Arabidopsis during senescence have identified many transporters, transcription factors, and other senescence associated genes that are up or down regulated (Buchanan-Wollaston et al., 2003, 2005; Van Der Graaff et al., 2006; Balazadeh et al., 2008). However, their correlation with specific changes in minerals or N remobilization is still incomplete. Our overall goal in this study was to identify and classify switchgrass transcripts into mineral transporter gene families and quantify their expression over the life cycle in different tissues as a first step to finding correlations between gene expression and mineral translocation through tissues. This will allow focused future studies to pinpoint the specific roles of individual genes during plant development.

### Discovery of potential switchgrass transporters

In mining the switchgrass genome for mineral transporter gene family members we found a total of 520 genes belonging to 40 different classes in the current annotation of the switchgrass genome. The number of switchgrass genes was approximately twice as many as identified in the annotated genomes of *Sorghum bicolor* (274) and *Setaria italica* (281) (Table 1). Our results indicate that for the most part, the switchgrass genome (tetraploid, A and B genomes) contained about twice as many genes in each class (Table 1), although some exceptions were noticed. For example, four putative copper transporters (*COPT*) (Pilon et al., 2009) were identified in the switchgrass genome, as compared to 1 each in sorghum and *Setaria*. Likewise, 7 potassium transporters (*HAKs*) (Grabov, 2007) were found in Sorghum and *Setaria*, whereas 9 putative *HAKs* were identified in the switchgrass genome. As anticipated, the switchgrass genome contained large numbers (>10 genes per genome) for many classes of transporters including those for nitrate, phosphate, S, K, Mg, and putative peptide/nitrate transporters.

**Table 1:** **Bioinformatic analysis of switchgrass genome for mineral transporter classes**.

	**Identifed transporters from genome mining**				
**Class**	**Descriptions**	**In ref**	**Pvi0**	**Sb**	**Si**
ACA	Ca2+-transporting ATPase	8	22	13	13
AKT/KAT	Shaker family K+ ion channel	9	18	12	11
AMT	Ammonium transporter	6	13	8	8
CAX	Cation/proton exchanger	7	16	7	8
CHX	Cation/H(+) antiporter	28	26	17	18
COPT	Copper transporter	5	4	1	1
FRO	Ferric reduction oxidase	8	1	1	1
FRU	ER-like iron deficiency-induced TF	1	2	1	2
HKT	Sodium transporter	1	5	3	4
HMA	Heavy metal ATPase	8	18	8	8
IREG	Iron regulated protein	3	6	3	3
IRT	Fe(2+) transport protein	3	1	0	0
KEA	K(+) efflux antiporter	6	9	4	4
KUP	Potassium transporter	13	33	22	21
LSI	Silicon transporter (from Hv)	2	5	2	2
MHX	Magnesium/proton exchanger	1	4	1	1
MOT	Molybdate transporter	2	5	1	2
MRS	Magnesium transporter	11	20	10	9
MTP	Metal tolerance protein	1	2	1	1
NAS	Nicotianamine synthase 1	4	4	3	3
NAXT	Nitrate excretion transporter	1	3	1	3
NHX	Sodium/hydrogen exchanger	8	15	7	7
NRAMP	Metal transporter	6	16	6	6
NRT	Nitrate transporter	16	35	18	19
NTRm	Misc NTR-class (major facilitator)	16	32	15	17
OPT	Oligopeptide transporter	9	15	9	8
PHO	Phosphate transporter	11	9	4	4
PHT	Inorganic phosphate transporter	19	45	22	24
PNT	Putative peptide/nitrate transporters	23	48	25	23
PTR	Peptide transporter	5	11	8	9
SULTR	Sulfate transporter	12	23	11	11
TPC	Two pore calcium channel protein	1	1	1	1
TPK	Ca-activated outward-rectifying K channel	1	7	2	3
VIT	Vacuolar iron transporter	1	4	2	2
YSL	Metal-nicotianamine transporter	8	15	11	13
ZIP	Zinc transporter	13	27	14	11
	Total	277	520	274	281

*In ref, Genes annotated as having mineral transporter function in the Arabidopsis thaliana genome; Pvi0, Draft 0.0 switchgrass genome; Sb, Sorghum bicolor; Si, Setaria italica*.

### Differential regulation of transporter genes in switchgrass tissues over development

**Figure 1:**
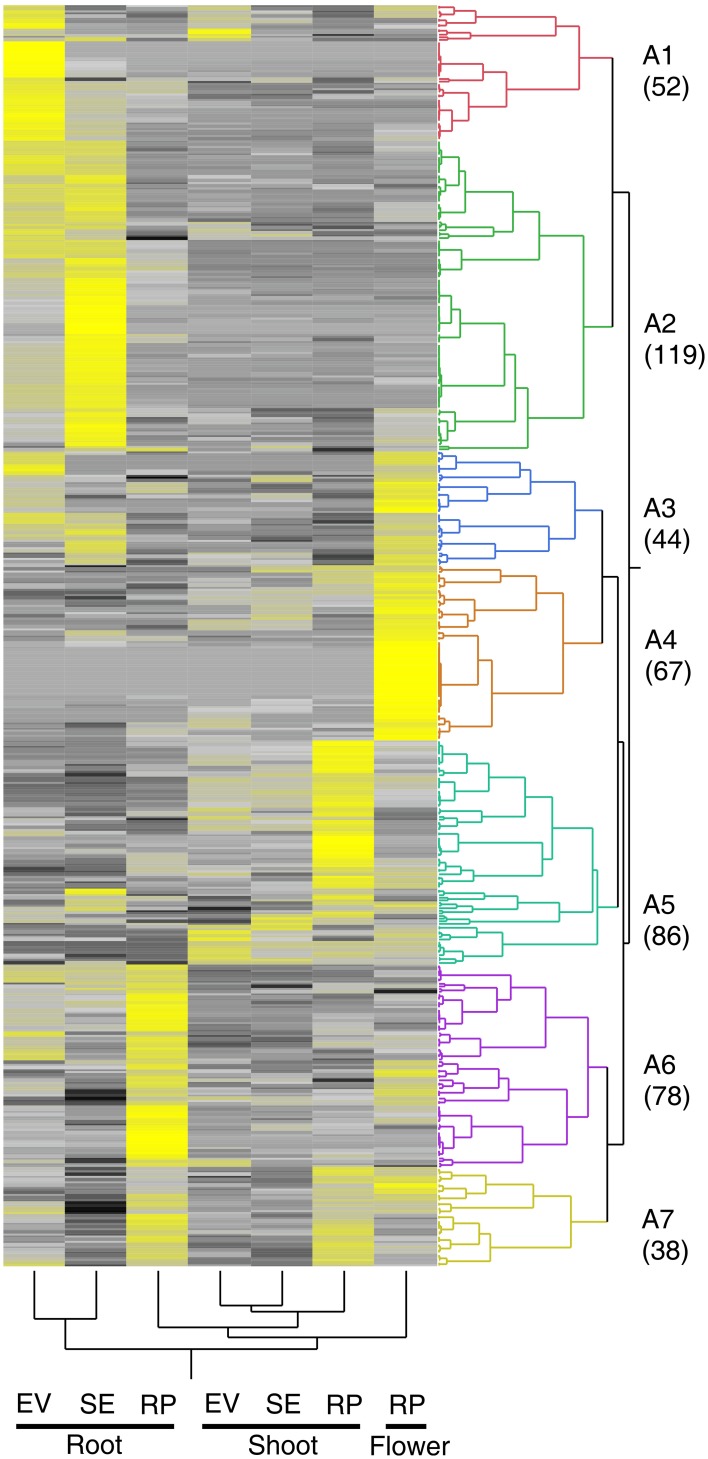
**Two-Way clustered expression profile map of genes of switchgrass nutrient transporters present in different tissues of cv Alamo plants grown in a greenhouse**. Stages of plant development are early vegetative (EV), stem elongation (SE), and reproductive (RP) are as described in these datasets. Yellow indicates high abundance, gray is intermediate and black is low or negligible abundance. The clusters are labeled as A1 through A7. The numbers in parentheses under each cluster indicate the total number of transporter identified within each cluster. The appropriate SRA identification numbers for these individual NGS files are SRX057826, SRX057827, SRX057828, SRX057829, SRX057830, SRX057831, and SRX057834.

Both tissue and temporal specificity in the expression of putative transporter genes in switchgrass was observed. Transcriptome datasets generated for greenhouse grown switchgrass cv Alamo (Figure 1) were mined for the relative abundance of transcripts for transporters shown in Table 1. There appeared to be both tissue and stage specific expression for many transporter genes at the three stages of harvest, early vegetative, shoot elongation and reproductive (Figure 1). Most transporter transcripts had different apparent abundances over plant developmental stages for the roots and shoots, and in flowers at reproductive stage (Figure 1). In roots, a cluster of transporters with high transcript counts were observed at the early vegetative stage of harvest (cluster A1). Several genes associated with this cluster were downregulated at the shoot elongation stage, and a larger cluster of transcripts were upregulated in roots at the shoot elongation stage (Figure 1; cluster A2). At the reproductive stage of plant growth, a new set of transporter genes was more abundant in the roots (cluster A6), and there was an apparent downregulation of many of the genes present in greater abundance at the early vegetative and shoot elongation stages of plant growth. A few genes appeared to be upregulated at the early vegetative stage in shoots as compared to roots. At the shoot elongation stage of plant development, most transporter genes were less abundant in shoots as compared to roots, and also less abundant than in shoots at the early vegetative stage (Figure 1). Interestingly, transcripts for a cluster of transporter genes had higher abundance in reproductive stage shoots (cluster A5). These included genes that appeared to be primarily expressed in shoots and some that overlapped with roots and reproductive structures. Reproductive tissues contained greater levels of transcripts for a cluster of transporter genes that were less abundant in roots or shoots (Figure 1, cluster A4).

**Figure 2:**
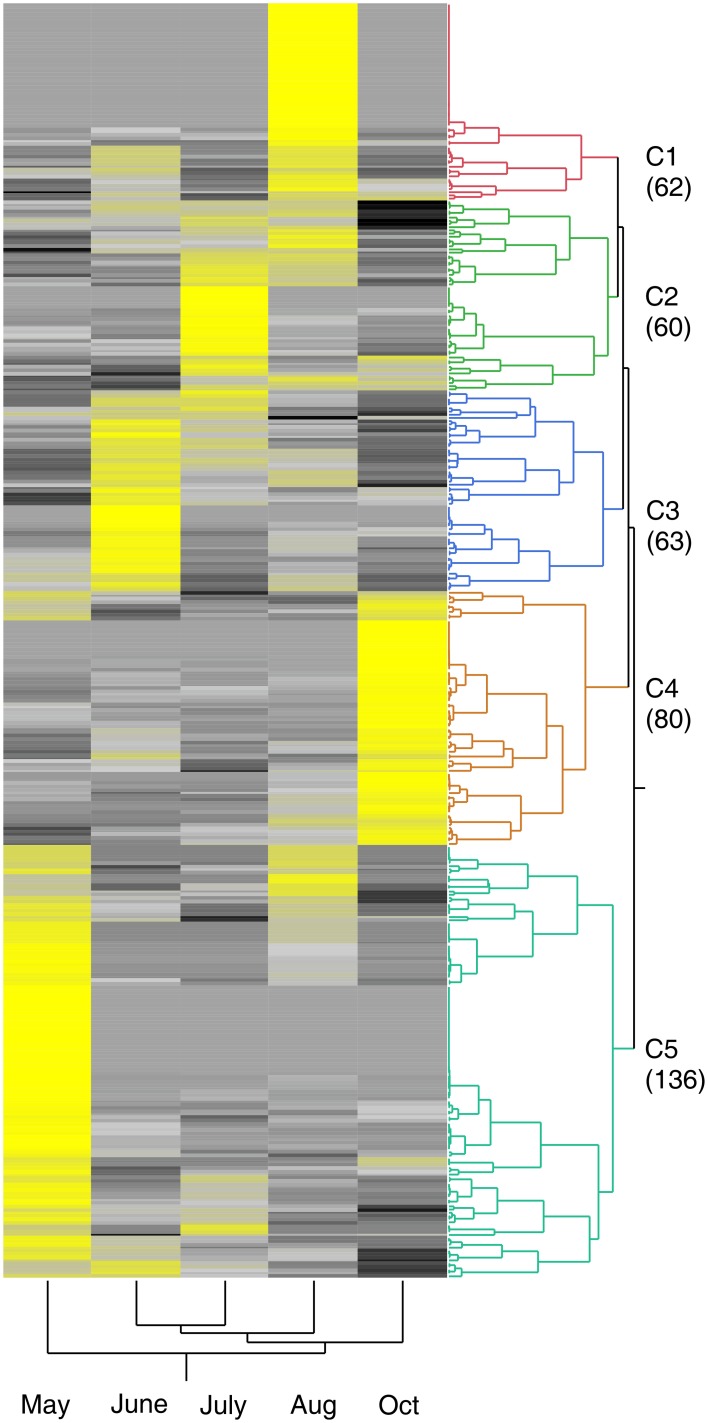
**Two-Way clustered expression profile map of genes of switchgrass nutrient transporters present in crown and rhizome tissues of field grown cv Summer plants harvested at different times during the growing season**. Yellow indicates high abundance, gray is intermediate and black is low or negligible abundance. The clusters are labeled as C1 through C5. The numbers in parentheses under each cluster indicate the total number of transporter identified within each cluster. The appropriate SRA identification numbers for these individual NGS files are SRX257007, SRX257030, SRX257031, SRX102934, and SRX257032.

We next evaluated expression profiles of several transporter gene families in field grown crown and rhizome tissues from cv Summer plants at different stages during a growing season. Of the 520 total mineral transporter genes identified in the switchgrass genome, transcripts for 401 mineral transporter genes were detected in the crown and rhizome datasets (see Table 1). As observed for the greenhouse grown cv Alamo datasets, some gene clusters were up- or downregulated at certain harvest dates (Figure 2). Some transcripts that were abundant early in the growing season (spring green up; May cluster C5) were less abundant later, suggesting that these genes are important for rapid growth in the spring. Enrichment of specific transporter classes (GO-terms) was not observed in these clusters. Other subsets of transporter genes were strongly upregulated during the periods of active shoot and rhizome growth (June and July, clusters C2 and C3), suggesting that genes in this cluster are important as plants continue to grow and progress to the reproductive development stage. At the July harvest plants were heading, with inflorescences visible at the top of the shoots. Although some of these genes were apparently being transcribed at continued high rates, a new cluster of transporter genes was upregulated at the August harvest date (Figure 2, cluster C1), when the plants were nearing physiological maturity, suggesting that these genes could be important for moving minerals to developing seeds. In crowns and rhizomes obtained from plants after a leaf killing frost in October, many of the transporter genes that were upregulated at the earlier harvest dates had decreased, whereas a new cluster of genes had increased transcript counts (cluster C4). This is a particularly interesting pattern, as these transporters are likely to be important for mineral storage or translocation of minerals to perennial storage tissues such as roots and rhizomes.

Comparison of the gene members for each cluster in the two 454 transporter datasets showed significant overlap. Approximately 40% of the genes expressed early in the growing season in crown and rhizomes (clusters C5 and C3) are also found expressed in roots during the early vegetative and shoot elongation stages in Alamo (clusters A1, A2, and A3). Similarly, approximately 30% of the genes expressed during the reproductive period in crowns and rhizomes (clusters C2 and C1) are expressed in Alamo roots during the reproductive stage (clusters A6 and A7). These metadata analyses from both greenhouse grown cv Alamo and field grown cv Summer plants indicated that nutrient transporters were transcriptionally controlled at the tissue level and expression was influenced by the developmental stage of the plant. It will be interesting and useful to compare the gene expression data to changes in mineral concentrations over the seasonal growth and senescence of switchgrass.

### Expression profiles of selected mineral transporter gene families

**Figure 3:**
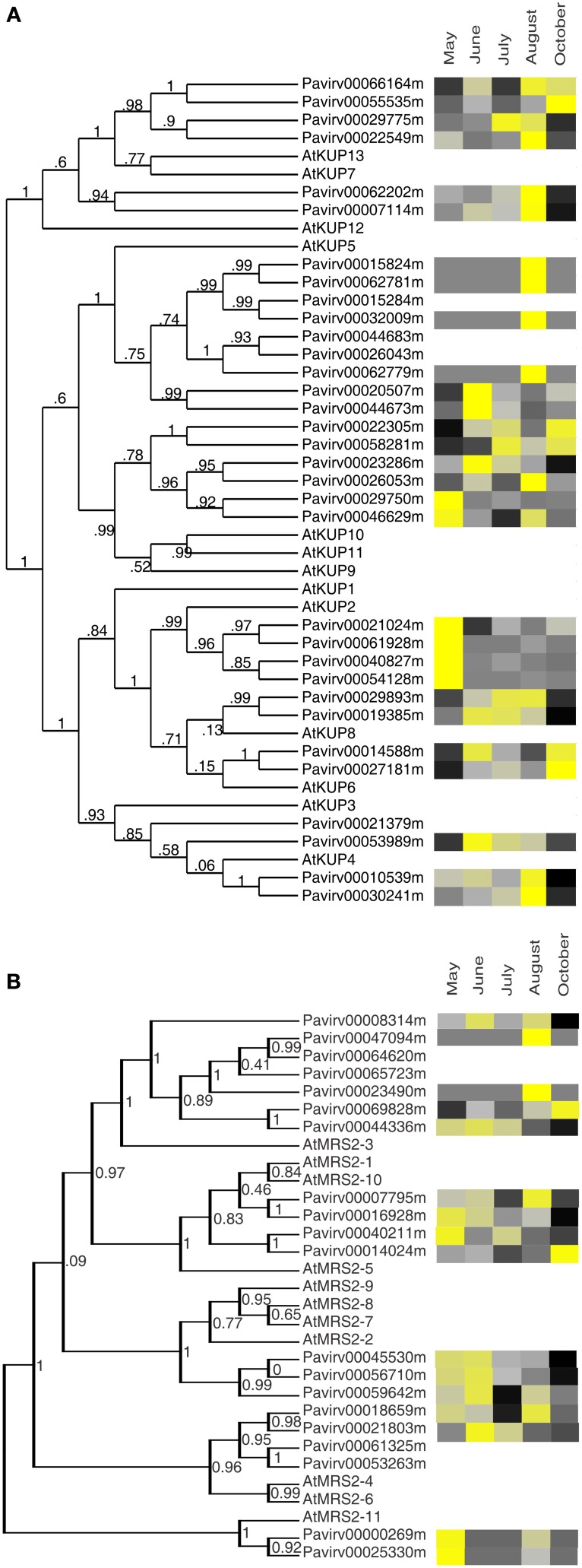
**Cladogram of the phylogentic relationships between switchgrass transporter genes to *Arabidopsis thaliana* orthologs and their expression profiles in crowns and rhizomes of field grown cv Summer plants. (A)** Potassium transporters (KUP) **(B)** Magnesium transporters (MRS). Other details are as described for Figure 2.

Phylogenetic relationships and expression levels for different classes of transporter genes were analyzed in crown and rhizome datasets. *HAK/KUP/KT* genes encode K^+^/H^+^ symporters (Szczerba et al., 2009) and are associated with the uptake of K^+^ into roots and efflux from vacuoles (Rodriguez-Navarro and Rubio, 2006). *KUP* genes are involved with a number of different aspects of plant development (Grabov, 2007), and expression of *KUP* genes throughout the plant (Szczerba et al., 2009) indicates roles in many tissue and cell types. A total of 33 *KUP* genes were found in our scan of the switchgrass genome, and transcripts ascribable to 29 of these genes were expressed in the crowns and rhizomes of field grown cv Summer plants (Figure 3A). Switchgrass and *Arabidopsis thaliana* annotated *KUP* sequences were separated into six clades. Two genes that share similarities to *AtKUP4, Pavirv00030241* and *Pavirv00010539*, were most highly expressed in the August harvest. Transcripts for switchgrass *KUPs* falling in the clade with *AtKUP2* were all overrepresented at the onset of spring growth. For the other *KUPs*, gene expression within clades was variable, although patterns associated with specific harvest dates were evident. For example, all the switchgrass *KUPs* with sequence similarities to *AtKUP7*/13 proteins were overrepresented in the August or November harvests, whereas transcript abundances for the larger clade of switchgrass *KUPs* with protein sequence similarities to *AtKUP5/10* were more variable (Figure 3A).

Magnesium is transported by members of the MRS2/MGT family, with expression in Arabidopsis noted in roots, leaves and senescing leaves, flowers, and pollen (Waters, 2011), and subcellular localizations in plasma membrane, tonoplast, mitochondria, ER and chloroplast. Most of the Mg in leaves is associated with ribosomes involved in protein synthesis, with the majority of the remaining fraction associated with chlorophyll (Karley and White, 2009). As such, it is not surprising that transcripts of several *MRS2* magnesium transporters were most abundant during the active shoot growth phase (May–July) (Figure 3B). However, four and two genes were highly expressed in the August and October harvests, respectively, suggesting that Mg or related mineral transport was active in the crowns and rhizomes at a time when the above ground parts of the plants were senescing (August) or fully senescent (October). These changes could arise potentially from Mg transported from roots and/or sequestration of Mg into different cellular compartments of the rhizome. It is conceivable that tiller buds and other meristematic tissues present on these rhizomes are metabolic sinks, and transport processes are associated with the continued delivery of nutrients to these critical organs.

**Figure 4:**
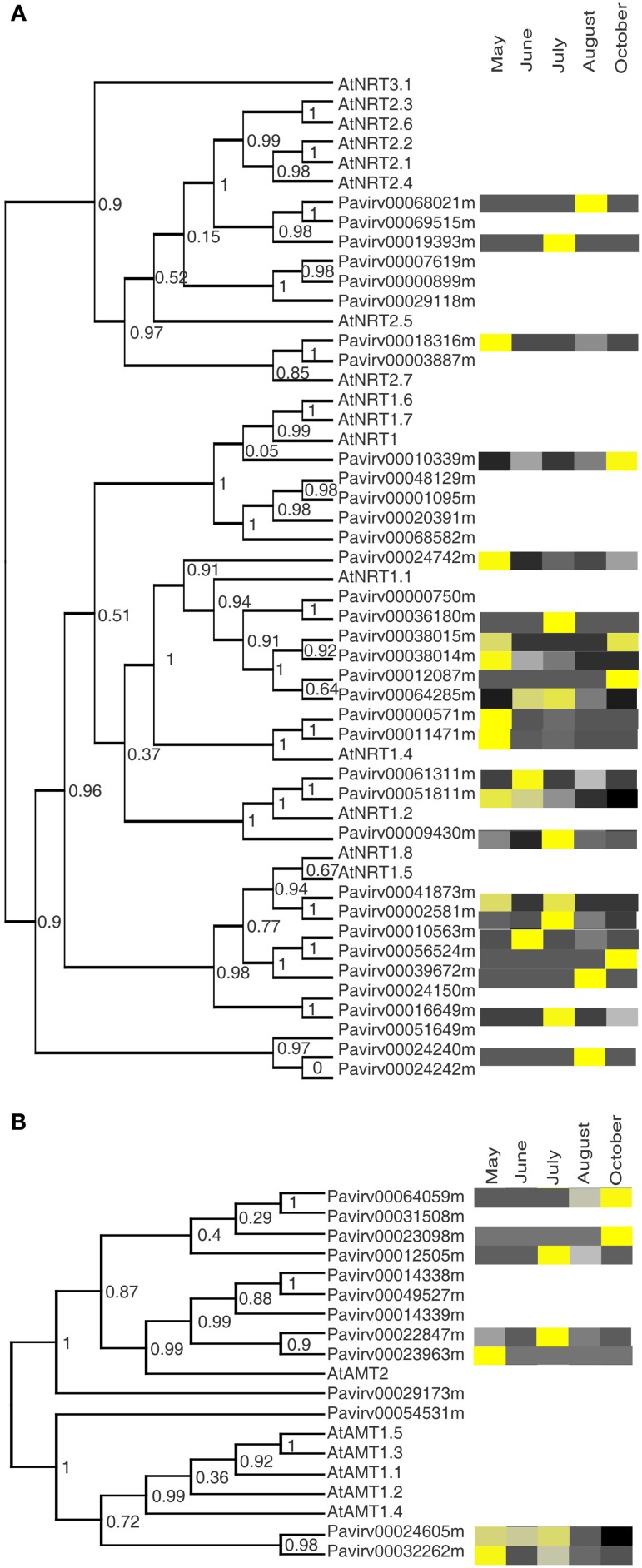
**Cladogram of the phylogentic relationships between switchgrass transporter genes to *Arabidopsis thaliana* orthologs and their expression profiles in crowns and rhizomes of field grown cv Summer plants. (A)** Nitrate transporters (NRT) **(B)** Ammonium transporters (AMT). Other details are as described for Figure 2.

Recycling of N in switchgrass is a major factor in sustainability and environmental impacts of production, and a number of studies have addressed this issue from production and genetic perspectives (reviewed by Schwartz and Amasino, 2013). Nitrate is a major source of N for plant roots, which is taken up by in part by nitrate transporters of the *NRT* family. Once nitrate is inside the plant, NRT transporters are also involved in xylem loading and unloading, phloem loading, and storage in vacuoles (Dechorgnat et al., 2011; Wang et al., 2012) and are essential for the translocation of plant defense compounds to the seeds (Nour-Eldin et al., 2012). Transcripts were detected for 22 putative switchgrass nitrogen transporters from a total of 35 identified in the switchgrass genome (see Table 1). The Arabidopsis *NRT2.4* gene is expressed in both the shoots and roots of nitrogen-starved plants (Kiba et al., 2012) and functions as a high affinity N transporter. Five switchgrass proteins with strong homology (*e*-values of 0 to 6 × 10^−170^) to Arabidopsis NRT2.4 were identified. Of these, transcripts for *Pavirv00019393* (Figure 4A) were most abundant in the July harvest when the plants had reached anthesis, whereas *Pavirv00068021* was overexpressed at the August harvest, when seeds were nearing physiological maturity. A majority of the other switchgrass *NRTs* (14) were most abundantly expressed during the active phase of shoot and rhizome growth (May–July harvests; Figure 4A). The other 6 *NRTs* were overexpressed in the August and October harvests. *Pavirv00010339* had higher expression in crowns and rhizomes of cv Summer plants at the October harvest date, and appears to be orthologous to the Arabidopsis NRT1.6 and 1.7 proteins. NRT1.7 is a low-affinity nitrate transporter involved in source to sink mobilization of nitrate via the phloem (Fan et al., 2009). In a similar manner, *Pavirv00039672*, with homology to the Arabidopsis NRT1.5/NRT1.8 transporters, is also upregulated in the October harvest (Figure 4A). Arabidopsis NRT1.5 modulates the allocation of nitrate to the roots to mediate stress responses in concert with NRT1.8 and other proteins (Chen et al., 2012).

Ammonium is an additional source of N for plant growth. Ammonium is taken into roots by AMT1 or AMT2 family transporters (Ludewig et al., 2007). Ammonium is also generated in tissues by reduction of nitrite or breakdown of amino compounds, some of which are remobilized during leaf senescence, and may need to be transported into chloroplasts for reassimilation. In Arabidopsis (Ludewig et al., 2007) and poplar (Couturier et al., 2007) *AMT* genes are expressed in roots, shoots, and flower structures. Switchgrass ammonium transporters were separable into four clades based on Arabidopsis (AtAMT) protein sequences (Figure 4B). Of the 13 AMT sequences in the switchgrass genome, transcripts for 7 genes were identified in the crown and rhizome. Five were more abundantly expressed during the active growing phases (May-July). Two genes overexpressed in crowns and rhizomes at the October harvest, *Pavirv00064059* and *Pavirv00023098* were orthologs of AtAMT2, which is a high affinity ammonium transporter in both shoots and roots (Sohlenkamp et al., 2002).

**Figure 5:**
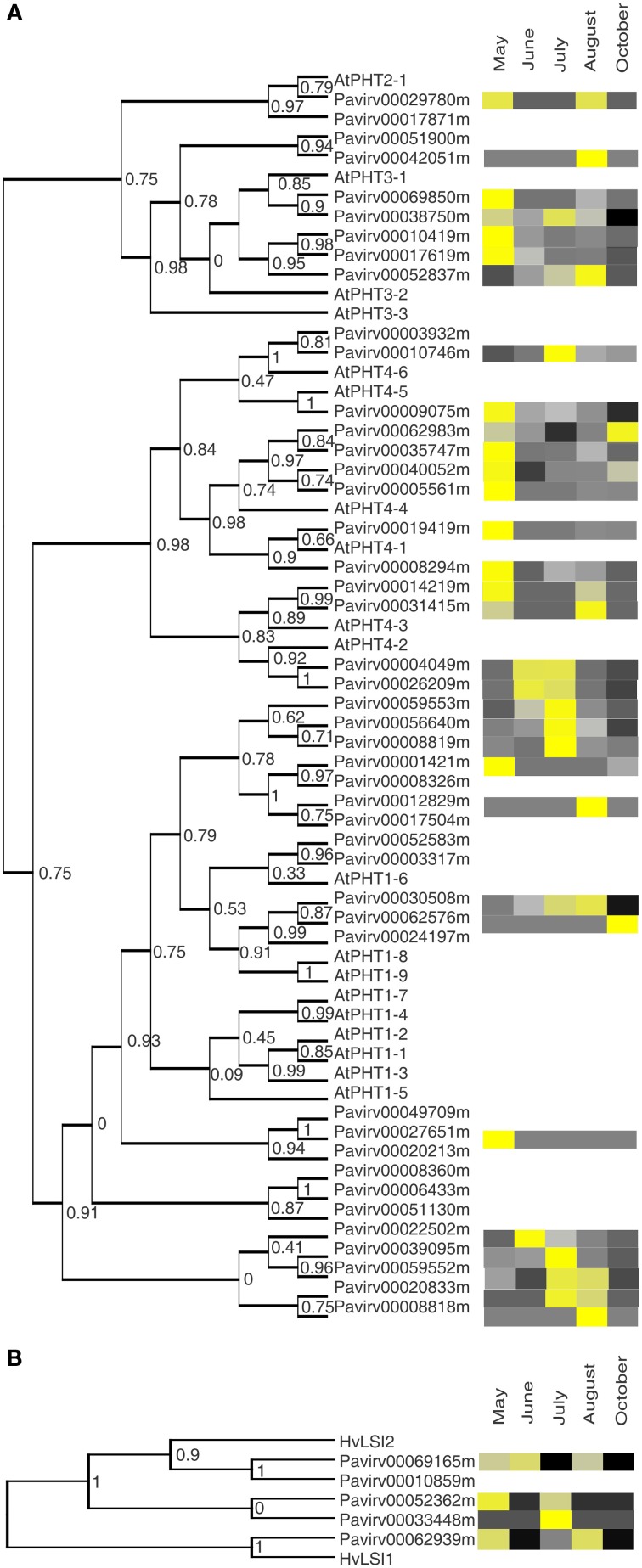
**Cladogram of the phylogentic relationships between switchgrass transporter genes to *Arabidopsis thaliana* (PHT) and barley (Hordeum vulgare; LSi) orthologs and their expression profiles in crowns and rhizomes of field grown cv Summer plants. (A)** Phosphate transporters (PHT) **(B)** Silicon transporters (LSi). Other details are as described for Figure 2.

In Arabidopsis, phosphate transporters of the PHT family are classified into 4 subfamilies (Liu et al., 2011) all of which had homologs in switchgrass. *PHT1* genes are primarily expressed in roots, where they are thought to take up phosphate from soil or mycorrhizal fungi, but are also expressed in leaves and pollen. *PHT2;1* is expressed primarily in the leaves and is thought to transport phosphate into leaves (Daram et al., 1998). PHT3 proteins are found in a variety of cellular membranes including mitochondria (Zhu et al., 2012). *PHT4* genes are mainly targeted to the plastids or Golgi (Guo et al., 2008) and are expressed in both roots and leaves. In rice (Liu et al., 2011) and Arabidopsis (Nussaume et al., 2011) *PHT* genes were expressed in numerous tissue types during the life cycle. Transcripts for ~71% of the total *PHT* genes in the switchgrass genome were found in the crown and rhizome of field grown switchgrass. Expression of the *PHTs* predominantly tracked with active growth phases of the plant (May–July harvests), although a smaller subset was upregulated in tissues harvested near physiological maturity (August, Figure 5A). Many of these genes clustered with Arabidopsis protein sequences belonging to the PHT3-2, 4-1, and 4-4 genes. These phosphate transporters are thought to be involved in a number of plant processes involving the shuttling of P_i_ across plant compartments. In crowns and rhizomes, this could involve both the acquisition and transport of phosphate from the soil to the developing shoots during the growing season as well as potentially in the redistribution of P_i_ at the end of the growing season. Notably, transcripts for *Pavirv00062983* (orthologous to *AtPHT4-4*) were upregulated in tissues obtained after a killing frost (October; Figure 5A). AtPHT4 has been implicated in the movement of P_*i*_ between the cytosol and plastids (Guo et al., 2008).

Silicon is important for resistance to abiotic and biotic stress in grasses (Ma et al., 2011; Nabity et al., 2012). About 90% of Si taken up by roots is transported into shoots (Ma and Takahashi, 2002). Lsi1 and Lsi2 are required for uptake (Ma et al., 2006, 2007), and Lsi6 is required for Si transport throughout leaves (Yamaji et al., 2008) and through stem nodes (Yamaji and Ma, 2009). To move from roots to leaves, Si would have to pass through crown tissue. Five genes putatively code for silicon transporters in switchgrass, and transcripts were detected for all five genes in the crown and rhizome tissues (Figure 5B). In contrast to other transporters, silicon transporters genes were essentially downregulated as the growing season progressed. Expression patterns of these putative switchgrass Si transporters are consistent with the movement of Si from the soil to the shoots. The downregulation of all of these Si-transporter genes possibly resulted from death of the shoots (October harvest, Figure 5B).

**Figure 6:**
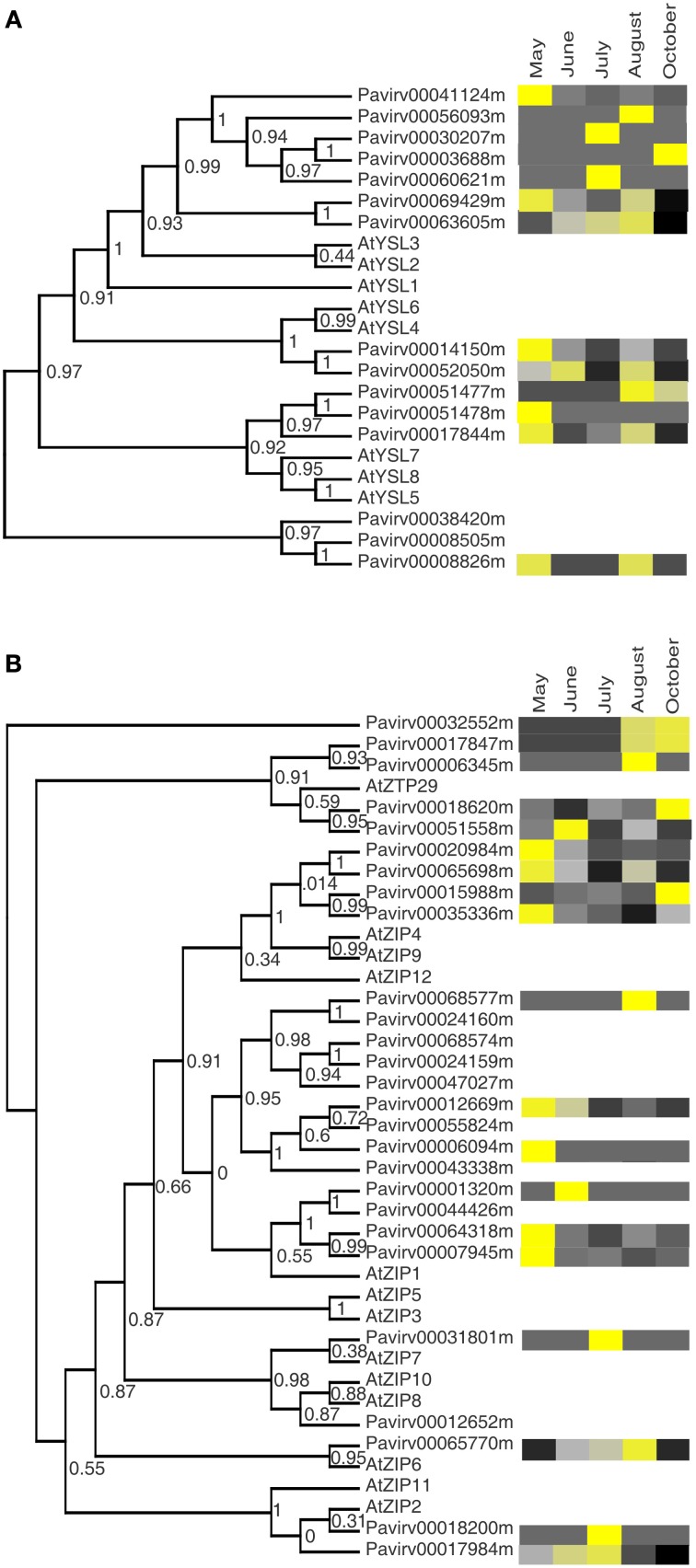
**Cladogram of the phylogentic relationships between switchgrass transporter genes to *Arabidopsis thaliana* orthologs and their expression profiles in crowns and rhizomes of field grown cv Summer plants. (A)** Yellow stripe-like transporters (YSL) **(B)** Zinc transporters (ZIP). Other details are as described for Figure 2.

We also analyzed the expression patterns of YSL and ZIP families of metal micronutrient transporters (Figures 6A,B). Yellow stripe-like (*YSL*) genes are related to the Yellow Stripe gene that encodes an Fe(III)-phytosiderophore uptake protein in maize roots (Curie et al., 2001). However, instead of functioning in uptake, YSL proteins carry out transport of nicotianamine-metal complexes within the plant, including Cu, Fe, Zn, and Mn (Waters et al., 2006; Curie et al., 2009; Ishimaru et al., 2010). Transcripts for all but two of 15 putative *YSLs* were found in the total crown and rhizome transcriptome dataset (Figure 6A). The expression patterns of these genes were similar to those for the *NRTs* and *PHTs*, in that certain genes were upregulated at different harvest dates, suggesting both developmental and tissue regulation. Notably, four of the *YSL* genes were at higher levels at the August harvests when minerals were being remobilized from shoots. Two Arabidopsis *YSL* genes have been implicated in remobilization of Cu, Fe, and Zn (Waters et al., 2006; Waters and Grusak, 2008). The *Pavirv00003688* transcript that is most closely related to Arabidopsis *YSL2* appeared to be strongly upregulated in the crown and rhizomes harvested in October (Figure 6A). Arabidopsis YSL2 probably functions in the lateral movement of Fe, Cu, and Zn in tissues (Didonato et al., 2004; Schaaf et al., 2005). Some of the switchgrass *YSL* paralogs showed a bimodal expression pattern, with higher transcript abundance in May and August harvests. The orthologous Arabidopsis genes *AtYSL4* and *YSL6* are key for iron homeostasis during plastid ontogeny, thereby modulating plant responses to iron availability (Divol et al., 2013).

Plant zinc/divalent metal transporters (ZIPs) are members of a relatively large group of related genes that participate in metal transport and homeostasis (Sinclair and Kramer, 2012), including Fe (Eide et al., 1996; Vert et al., 2009), Zn (Lin et al., 2009; Milner et al., 2013), and Mn (Milner et al., 2013), with transport across plasma or vacuolar membranes (Waters and Sankaran, 2011; Milner et al., 2013). Transcripts were detected for 19 of 27 switchgrass *ZIPs* in the crown and rhizome transcriptomes (Figure 6B). Two *ZIP* genes that are orthologous to Arabidopsis *ZIP2* and *ZIP7* were overexpressed in the July harvests. AtZIP2 is localized on the root plasma membrane and is thought to aid in the loading of Mn and Zn into the xylem (Milner et al., 2013). Since above ground growth in plants is active in July, it is possible that the switchgrass orthologs fill a similar role. Transcripts for nine *ZIPs* were more abundant in the early (predominately May) harvest. Among these were three genes orthologous to the Arabidopsis *ZIPs* 4, 9, and 12, which are induced by Zn deprivation (Jain et al., 2013). A related switchgrass gene (*Pavirv00015988*), however, was overexpressed in crowns and rhizomes at the end of the growing season (October; Figure 6B). Four switchgrass zinc transporter (*ZTP*) genes with homology to the Arabidopsis *ZTP29* were upregulated in crown and rhizome tissues later in the growing season (August and October). AtZTP29 is localized to the ER and induced in roots in response to salt stress, and is thought to play a role in the unfolded protein response (Wang et al., 2010).

**Figure 7:**
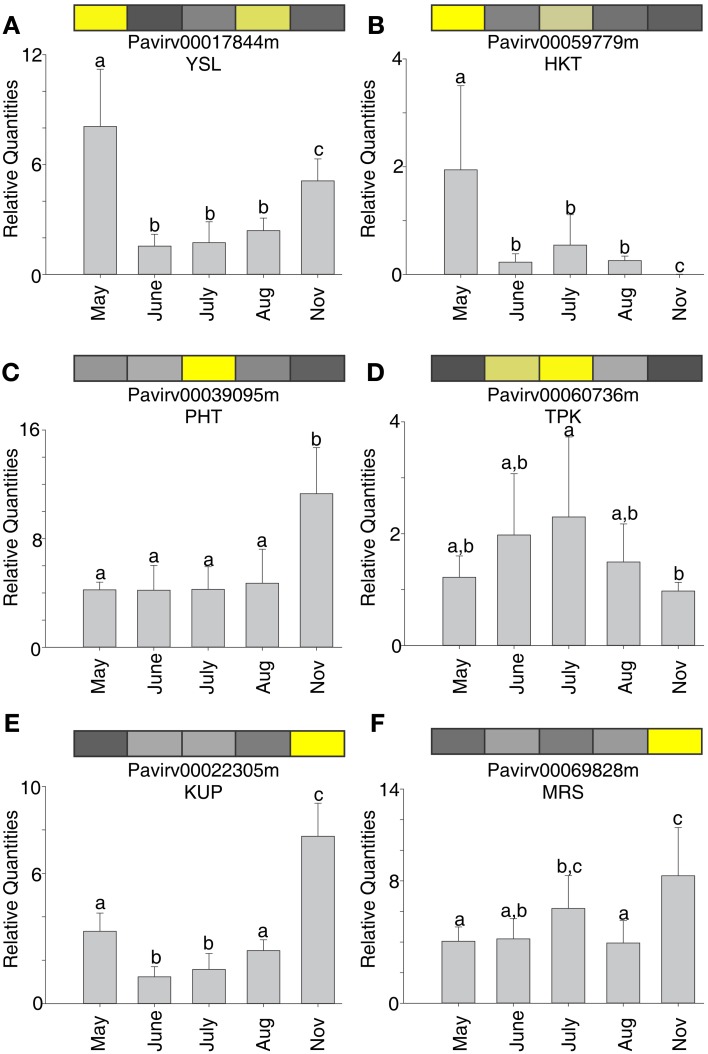
**Correspondence between expression profiles observed in NGS datasets to qRT-PCR data for six select switchgrass transporter genes. (A)** YSL; **(B)** HKT; **(C)** PHT; **(D)** TPK; **(E)** KUP; **(F)** MRS. Colored bar at the top of each panel is the expression profile for each gene observed in the 454 NGS dataset (see Figure 2). Yellow is high expression and black is low or negligible expression. Gray bars in each panel show the relative expression (±*SD*) for each individual gene as determined by qRT-PCR. Different letters above each bar was significantly different expression at *P* < 0.05. See text for more details.

The distinct temporal changes in transcript abundances in the 454 dataset were validated using qRT-PCR for arbitrarily selected genes that in the 454 datasets (See Figures 3–6) were at higher abundance at a specific harvest date. Six transporter genes and one reference gene were analyzed by qRT-PCR using RNA from field grown plants (Figure 7). In 5 out of 6 genes, transcript abundance by qRT-PCR agreed with the 454 expression datasets. Transcript abundance determined by the two methods corresponded closely for four genes (Figures 7B,D,E,F), where a simple regression of transcript counts at each harvest date to the relative quantities of abundance by qRT-PCR yielded *R*^2^-values between 0.69 and 0.97. For two others (Figures 7A,C), the *R*^2^-values were 0.37 **(A)** and 0.21 **(C)**. In the case of the *YSL* gene (Figure 7A), the highest abundance for both datasets was in May, but the abundances observed in the August and November harvest dates were different, resulting in the lower correlation coefficient. The *PHT* gene *Pavirv00039095* (Figure 7C) had a weak correlation coefficient between the 454 dataset (highest in July) and the qRT-PCR dataset (highest in November). Taken together, these data support the findings presented in Figures 3–6.

### Mineral dynamics in crown and rhizome

**Figure 8:**
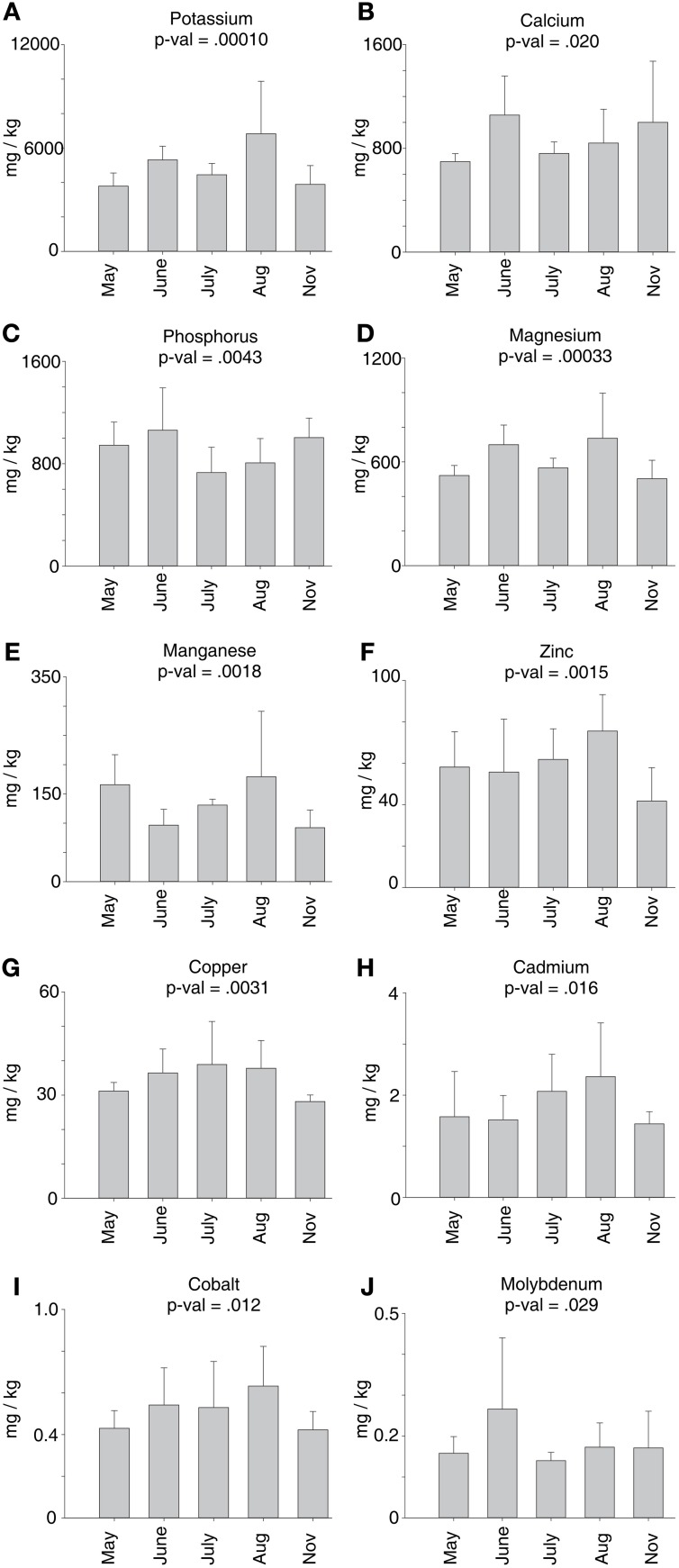
**Mineral concentrations in crowns and rhizomes of field grown cv Summer plants across harvest dates**. Each panel shows the change in mineral concentration for a specific mineral. Analysis for each time point consisted of two technical replicates from each of three biological replicates (*n* = 6). Average abundances with standard deviation error bars are shown in this figure. *P*-values were calculated for each mineral using Single-Factor ANOVA. **(A)** Potassium; **(B)** Calcium; **(C)** Phosphorus; **(D)** Magnesium; **(E)** Manganese; **(F)** Zinc; **(G)** Copper; **(H)** Cadmium; **(I)** Cobalt; **(J)** Molybdenum.

Mineral concentrations were analyzed in crown and rhizome tissues. Of the 16 minerals analyzed, As, Fe, Na, Ni, S, and Se levels did not change significantly across harvests. The remaining 10 minerals with significant difference between any two harvest dates are shown in Figure 8. Excluding P and Ca, all the other minerals had lowest concentrations in crowns and rhizomes harvested in November. For K, Mg, Mn, Zn, Cu, Cd, and Co, maximal levels were detected at the August harvest, when the shoots were at an advanced stage of senescence. Highest levels of Mo were found in rhizomes harvested in June. A bimodal pattern of mineral concentrations was seen for K, Ca, P, Mg, and Mn. For K, Mg, and Mn, maximal concentrations were observed in tissues harvested in May (Mn) and June (period of active growth) and in August (shoot senescence). For Ca and P maximal amounts were observed for the June and November harvests, respectively (Figure 8). These fluctuating concentrations may reflect the passage of minerals through the crowns seasonally. Cu, K, and Zn are minerals known to be remobilized, and were lowest during shoot dormancy when recycled minerals would be stored in roots. Alternatively, since large fluctuations were not observed, mineral concentrations may reflect demands of the tissues themselves for growth and metabolism.

## Conclusions and future directions

This research represents a first step in the characterization of mineral transporter genes and associating their expression in a perennial grass. As more data for mineral dynamics becomes available, a clear picture of the genes needed for translocation to shoots in spring and to storage tissues in the fall will emerge. We have identified a number of mineral transporter genes with seasonal expression patterns that give clues to the biology of crown and rhizome tissues as a gateway between shoot growth and mineral storage and uptake. Likewise, this tissue is also a recipient of nutrients remobilized from senescing shoots. We anticipate that as additional transcriptomic and mineral datasets from other switchgrass tissues become available, these resources will be a valuable tool for plant breeders to improve production and sustainability of switchgrass.

### Conflict of interest statement

The authors declare that the research was conducted in the absence of any commercial or financial relationships that could be construed as a potential conflict of interest.
