# Supplementary material for: Global changes in mineral transporters in tetraploid switchgrasses (Panicum virgatum L.)
Source: Front Plant Sci. 2014 Jan 2;4:549. doi: 10.3389/fpls.2013.00549 (PMC3878055; doi:10.3389/fpls.2013.00549)
Supplement: Supplementary file 1 [file DataSheet1.DOCX]

Table S1. Primers used in the study.

| Gene | Class | Fordward Primer | Reverse Primer |
| --- | --- | --- | --- |
| Pavirv00017844m | YSL | TTTTGGTTGCCTATGTGGTGGCG | AAGCGCCGAACACGAAGATGG |
| Pavirv00059779m | HKT | TACACCACATTTTTGCCGGTGGC | GCTTCTGCTGCCACACGCTTC |
| Pavirv00039095m | PHT | TGACGGCCTTCATGCTCGGC | TTGGGCCCGAAGTTGGCGAAG |
| Pavirv00060736m | TPK | CCAATTGGGGCGTGTTTTTGCG | TTTGGCGAGCATTTTCTGCCGTC |
| Pavirv00022305m | KUP | TTTCACCGCGCTGTCATTGGTTG | AAGGCGACGGCAAACACAAGAG |
| Pavirv00069828m | MRS | ACGCGGGGAAGAGGAGCAAAATC | TTATTGCACCACACACGCAACCG |
| Pavirv00026367m | Ubq Ligase | TGCTCTTGCCTTTTTCTGCCTGC | TTATGCAGGGTGTTGCGCTGTG |
